# Global distribution of malocclusion traits: A systematic review

**DOI:** 10.1590/2177-6709.23.6.40.e1-10.onl

**Published:** 2018

**Authors:** Maged Sultan Alhammadi, Esam Halboub, Mona Salah Fayed, Amr Labib, Chrestina El-Saaidi

**Affiliations:** 1 Jazan University, College of Dentistry, Department of Preventive Sciences, Division of Orthodontics and Dentofacial Orthopedics (Jazan, Saudi Arabia).; 2 Ibb University, Faculty of Oral and Dental Medicine, Department of Orthodontics and Dentofacial Orthopedics (Ibb, Republic of Yemen).; 3 Jazan University, College of Dentistry, Department of Maxillofacial Surgery and Diagnostic Sciences (Jazan, Saudi Arabia).; 4 Cairo University, Faculty of Oral and Dental Medicine, Department of Orthodontics and Dentofacial Orthopedics (Cairo, Egypt).; 5 University of Malaya, Faculty of Dentistry, Department of Pediatric Dentistry and Orthodontics (Kuala Lumpur, Malaysia).; 6 Kyoto University, Graduate School of Medicine, Department of Global Health and Socio-epidemiology (Kyoto, Japan).

**Keywords:** Prevalence, Malocclusion, Global health, Population, Permanent dentition, Mixed dentition.

## Abstract

**Objective:**

Considering that the available studies on prevalence of malocclusions are local or national-based, this study aimed to pool data to determine the distribution of malocclusion traits worldwide in mixed and permanent dentitions.

**Methods:**

An electronic search was conducted using PubMed, Embase and Google Scholar search engines, to retrieve data on malocclusion prevalence for both mixed and permanent dentitions, up to December 2016.

**Results:**

Out of 2,977 retrieved studies, 53 were included. In permanent dentition, the global distributions of Class I, Class II, and Class III malocclusion were 74.7% [31 - 97%], 19.56% [2 - 63%] and 5.93% [1 - 20%], respectively. In mixed dentition, the distributions of these malocclusions were 73% [40 - 96%], 23% [2 - 58%] and 4% [0.7 - 13%]. Regarding vertical malocclusions, the observed deep overbite and open bite were 21.98% and 4.93%, respectively. Posterior crossbite affected 9.39% of the sample. Africans showed the highest prevalence of Class I and open bite in permanent dentition (89% and 8%, respectively), and in mixed dentition (93% and 10%, respectively), while Caucasians showed the highest prevalence of Class II in permanent dentition (23%) and mixed dentition (26%). Class III malocclusion in mixed dentition was highly prevalent among Mongoloids.

**Conclusion:**

Worldwide, in mixed and permanent dentitions, Angle Class I malocclusion is more prevalent than Class II, specifically among Africans; the least prevalent was Class III, although higher among Mongoloids in mixed dentition. In vertical dimension, open bite was highest among Mongoloids in mixed dentition. Posterior crossbite was more prevalent in permanent dentition in Europe.

## Resumo

**Objetivo:**

considerando-se que os estudos disponíveis sobre a prevalência das más oclusões são de base local ou nacional, esse estudo teve como objetivo reunir dados para determinar a distribuição dos tipos de má oclusão em uma escala global, nas dentições permanente e mista.

**Métodos:**

foi realizada uma busca eletrônica através das ferramentas de pesquisa do PubMed, Embase e Google Acadêmico, para reunir estudos publicados até dezembro de 2016 sobre a prevalência das más oclusões, tanto na dentição permanente quanto na dentição mista.

**Resultados:**

dos 2.977 estudos encontrados, 53 foram analisados. Na dentição permanente, a distribuição mundial das más oclusões de Classe I, II e III foi, respectivamente, de 74,7% [31 - 97%], 19,56% [2 - 63%] e 5,93% [1 - 20%]. Na dentição mista, a distribuição dessas más oclusões foi de 73% [40 - 96%], 23% [2 - 58%] e 4% [0,7 - 13%]. Em relação às más oclusões verticais, observou-se prevalência de 21,98% de sobremordida profunda e 4,93% de mordida aberta. A mordida cruzada posterior afetou 9,39% da amostra. Os africanos mostraram a maior prevalência de Classe I e mordida aberta na dentição permanente (89% e 8%, respectivamente) e na dentição mista (93% e 10% respectivamente), enquanto os caucasianos apresentaram a maior prevalência de Classe II na dentição permanente (23%) e na dentição mista (26%). A má oclusão de Classe III na dentição mista foi mais prevalente entre xantodermas.

**Conclusão:**

mundialmente, nas dentições mista e permanente, as más oclusões de Classe I de Angle são mais prevalentes do que as de Classe II, especificamente entre os africanos; a menos prevalente foi a Classe III, ainda que mais prevalente entre os xantodermas na dentição mista. Na dimensão vertical, as mordidas abertas foram mais prevalentes entre xantodermas na dentição mista. A mordida cruzada posterior apresentou maior prevalência na dentição permanente na Europa.

## INTRODUCTION

Angle introduced his famous classification of malocclusion in 1899.^1^ Now the World Health Organization estimates malocclusions as the third most prevalent oral health problem, following dental caries and periodontal diseases.^2^

Many etiological factors for malocclusion have been proposed. Genetic, environmental, and ethnic factors are the major contributors in this context. Certain types of malocclusion, such as Class III relationship, run in families, which gives a strong relation between genetics and malocclusion. Likewise is the ethnic factor, where the bimaxillary protrusion, for example, affects the African origin more frequently than other ethnicities. On the other hand, functional adaptation to environmental factors affects the surrounding structures including dentitions, bone, and soft tissue, and ultimately resulting in different malocclusion problems. Thus, malocclusion could be considered as a multifactorial problem with no specific cause so far.^3^

A search in the literature for studies on prevalence of malocclusion and related factors revealed that most of these epidemiological investigations were published between the 1940s and the 1990s. Thereafter, publications have been turned into focusing more on determination of treatment needs, treatment techniques and mechanisms, and treatment outcomes.^4^

Epidemiological studies play a pivotal role in terms of determining the size of the health problems, providing the necessary data and generating and analyzing hypotheses of associations, if any. Through these valuable information, the priorities are set and the health policies are developed.^5^ Hence, the quality of these epidemiological studies must be evaluated crucially and it will be valuable to pool their results, whenever possible.

In this regard, there has been a continuous increase in conducting critical analyses for the published epidemiological health studies. The aim behind this is to generate a more precise and trusted evidence on the health problem under investigation using strict criteria for quality analysis. However, few have been conducted in orthodontics. The objective of the current study, therefore, was to present a comprehensive estimation on the prevalence of malocclusion in different populations and continents.

## MATERIALS AND METHODS

### Search method

A literature search in PubMed, Embase, and Google Scholar search engines was conducted up to December 2016. The following search terms were used: ‘Prevalence’, ‘Malocclusion’, ‘Mixed dentition', and 'Permanent dentition’. In addition, an electronic search in websites of the following journals was conducted: Angle Orthodontist, American Journal of Orthodontics and Dentofacial Orthopedics, Journal of Orthodontics, and European Journal of Orthodontics. 

Studies that fulfilled the following criteria were included:

1) Population-based studies.
2) Sample size greater than 200 subjects.
3) Studies that evaluated malocclusion during mixed and/ or permanent dentitions.
4) Studies that used Angle's classification of malocclusion.
5) Studies that considered the following definitions of the specified malocclusion characteristics: “abnormal overjet” if more than 3mm; “reverse overjet” when all four maxillary incisors were in a crossbite; “abnormal overbite” if more than 2.5 mm (for deep bite) and if less than 0 mm (for open bite); and “posterior crossbite” when affecting more than two teeth. The malocclusion traits included were: Angle Classification (Class I / II / III), overjet (increased / reversed), overbite (deep bite / open bite), posterior crossbite, based on the above mentioned definitions for these traits.

A study was excluded if it was conducted in a clinical/hospital-based setting and/or targeted malocclusion prevalence in primary dentition or in a population with specific medical problem.

Characteristics of all studies^6^ ^-^ ^58^ analyzed were formulated similar to that used in analysis of epidemiological studies^59^ ^,^ ^60^ (Table 1). 

**Table 1:** Characteristics of the included studies.

No	Author	Year	Sample	Age	Gender	Country	Region	Race	Population
1	Massler and Frankel^6^	1951	2758	14-18	M=1238, F=1520	America	America	Caucasian	Schoolchildren
2	Goose et al.^7^	1957	2956	7-15	Not mentioned	Britain	Europe	Caucasian	Schoolchildren
3	Mills^8^	1966	1455	8-17	M=719, F=736	America	America	Caucasian	Schoolchildren
4	Grewe et al.^9^	1968	651	9-14	M=322, F=329	America	America	Caucasian	Community
5	Helm^10^	1968	1700	6-18	M=742, F=958	Denmark	Europe	Caucasian	Schoolchildren
6	Thilander and Myrberg^11^	1973	6398	7-13	M=3093, F=3305	Sweden	Europe	Caucasian	Schoolchildren
7	Foster and Day^12^	1974	1000	12	Not mentioned	Britain	Europe	Caucasian	Schoolchildren
8	Ingervall et al.^13^	1978	389	21-54	M=389, F=0	Sweden	Europe	Caucasian	Military service
9	Helm and Prydso^14^	1979	1536	14-18	Not mentioned	Denmark	Europe	Caucasian	Schoolchildren
10	Lee et al.^15^	1980	2092	17-21	M=1281, F=811	Korea	Asia	Mongoloids	Community
11	Gardiner^16^	1982	479	10-12	Not mentioned	Libya	Africa	Caucasian	Community
12	De Muňiz^17^	1986	1554	12-13	M=655, F=899	Argentine	America	Caucasian	Schoolchildren
13	Kerosuo et al.^18^	1988	642	11-18	M=340, F=302	Tanzania	Africa	Africans	Schoolchildren
14	Woon et al.^19^	1989	347	15-19	Not mentioned	China	Asia	Mongoloids	Community
15	Al-Emran et al.^20^	1990	500	14	M=500, F=0	Saudia	Asia	Caucasian	Schoolchildren
16	El-Mangoury and Mostafa^21^	1990	501	18-24	M=231, F=270	Egypt	Africa	Caucasian	Community
17	Lew et al.^22^	1993	1050	12-14	Not mentioned	China	Asia	Mongoloids	Schoolchildren
18	Tang^23^	1994	201	20	Not mentioned	China	Asia	Mongoloids	Community
19	Harrison and Davis^24^	1996	1438	7-15	Not mentioned	Canada	America	Caucasian	Community
20	Ng’ang’a et al.^25^	1996	919	7-15	M=468, F=451	Kenya	Africa	Africans	Community
21	Ben-Bassat et al.^26^	1997	939	6-13	M=442, F=497	Israel	Asia	Caucasian	Schoolchildren
22	Proffit et al.^27^	1998	14000	8-50	Not mentioned	America	America	Caucasian	Community
23	Dacosta^28^	1999	1028	11-18	M= 484, F=544	Nigeria	Africa	Africans	Community
24	Saleh^29^	1999	851	9-15	M=446, F=405	Lebanon	Asia	Caucasian	Schoolchildren
25	Esa et al.^30^	2001	1519	12-13	M=772, F=747	Malaysia	Asia	Mongoloids	Schoolchildren
26	Thilander et al.^31^	2001	4724	5-17	M=2371, F=2353	Colombia	America	Caucasian	Heath center
27	Freitas et al.^32^	2002	520	11-15	M=250, F=270	Brazil	America	Caucasian	Schoolchildren
28	Bataringaya^33^	2004	402	14	M=141, F=261	Uganda	Africa	Africans	Schoolchildren
29	Onyeaso^34^	2004	636	12-17	M=334, F=302	Nigeria	Africa	Africans	Schoolchildren
30	Tausche et al.^35^	2004	197	6-8	M=970, F=1005	Germany	Europe	Caucasian	Schoolchildren
31	Abu Alhaija et al.^36^	2005	1003	13-15	M=619, F=384	Jordan	Asia	Caucasian	Schoolchildren
32	Ali and Abdo^37^	2005	1000	7-12	M=501, F=499	Yemen	Asia	Caucasian	Schoolchildren
33	Behbehani et al.^38^	2005	1299	13-14	M=674, F=625	Kuwait	Asia	Caucasian	Schoolchildren
34	Ciuffolo et al.^39^	2005	810	11-14	M=434, F=376	Italy	Europe	Caucasian	Schoolchildren
35	Karaiskos^40^	2005	395	9	Not mentioned	Canada	America	Caucasian	Schoolchildren
36	Ahangar Atashi^41^	2007	398	13-15	Not mentioned	Iran	Asia	Caucasian	Community
37	Gelgör et al.^42^	2007	810	11-14	M=1125, F=1204	Turkey	Europe	Caucasian	Health center
38	Jonsson et al.^43^	2007	829	31-44	M=342, F=487	Iceland	Europe	Caucasian	Schoolchildren
39	Josefsson et al.^44^	2007	493	12-13	Not mentioned	Sweden	Europe	Caucasian	Schoolchildren
40	Ajayi^45^	2008	441	11-18	M=229, F=212	Nigeria	Africa	Africans	Schoolchildren
41	Mtaya^46^	2008	1601	12-14	M=632, F=969	Tanzania	Africa	Africans	Schoolchildren
42	Borzabadi-Farahani et al.^47^	2009	502	11-14	M=249, F=253	Iran	Asia	Caucasian	Schoolchildren
43	Daniel et al.^48^	2009	407	9-12	M= 191, F=216	Brazil	America	Caucasian	Schoolchildren
44	Šidlauskas and Lopatienė^49^	2009	1681	7-15	M=672, F=1009	Lithuania	Europe	Caucasian	Schoolchildren
45	Alhammadi^50^	2010	1000	18-25	M=500, F=1000	Yemen	Asia	Caucasian	Schoolchildren
46	Bhardwaj et al.^51^	2011	622	16-17	M= 365, F=257	India	Asia	Caucasian	Schoolchildren
47	Nainani and Relan^52^	2011	436	12-15	M= 224, F=212	India	Asia	Caucasian	Schoolchildren
48	Bugaighis et al.^53^	2013	343	12-17	M=169, F=174	Libya	Africa	Caucasian	Schoolchildren
49	Kaur et al.^54^	2013	2400	13-17	M=1192, F=1208	India	Asia	Caucasian	Schoolchildren
50	Reddy et al.^55^	2013	2135	6-10	M=1009, F=1126	India	Asia	Caucasian	Schoolchildren
51	Bilgic F et al.^56^	2015	2329	12.5-16.2	M=1125, F=1204	Turkey	Europe	Caucasian	Schoolchildren
52	Gupta et al.^57^	2016	500	12-17	M=1125, F=1204	India	Asia	Caucasian	Schoolchildren
53	Narayanan et al.^58^	2016	2366	10-12	M=1281, F=1085	India	Asia	Caucasian	Schoolchildren

M = male; F = female.

Critical appraisal of the included studies was done based on a modified version of STROBE checklist^61^ ^,^ ^62^ comprising seven items related to: study design, study settings, participants criteria, sample size, variable description, and outcome measurements. The quality of the studies was categorized into weak (≤ 3), moderate (4 or 5) and high quality (≥ 6), as described in Table 2. 

**Table 2:** STROBE -based quality analysis of the included studies.

No	Author	Study design	Setting	Participants	Sample size	Variables description	Outcome measurement	Statistical analysis	Total score
1	Massler and Frankel^6^	✓	✓	✓	X	✓	✓	✓	5
2	Goose et al.^7^	X	✓	✓	X	X	✓	✓	4
3	Mills^8^	X	✓	✓	X	✓	✓	✓	5
4	Grewe et al.^9^	X	✓	✓	X	✓	✓	✓	5
5	Helm^10^	✓	✓	✓	X	✓	✓	✓	6
6	Thilander and Myrberg^11^	✓	✓	✓	X	✓	✓	✓	6
7	Foster and Day^12^	X	X	✓	X	✓	✓	✓	4
8	Ingervall et al.^13^	X	X	✓	X	✓	✓	✓	4
9	Helm and Prydso^14^	X	✓	✓	✓	✓	✓	✓	6
10	Lee et al.^15^	X	✓	✓	X	✓	✓	✓	5
11	Gardiner^16^	X	✓	✓	X	✓	✓	✓	5
12	De Muňiz^17^	X	✓	✓	X	X	✓	✓	4
13	Kerosuo et al.^18^	X	✓	✓	X	✓	✓	✓	5
14	Woon et al.^19^	X	✓	✓	X	✓	✓	✓	5
15	Al-Emran et al.^20^	X	✓	✓	X	X	✓	✓	4
16	El-Mangoury and Mostafa^21^	X	✓	✓	X	X	✓	✓	4
17	Lew et al.^22^	X	✓	✓	X	✓	✓	✓	5
18	Tang^23^	X	✓	✓	X	✓	✓	✓	5
19	Harrison and Davis^24^	X	✓	✓	X	✓	✓	✓	5
20	Ng’ang’a et al.^25^	X	✓	✓	✓	X	✓	✓	6
21	Ben-Bassat et al.^26^	X	✓	✓	X	✓	✓	✓	5
22	Proffit et al.^27^	✓	✓	✓	X	✓	✓	✓	6
23	Dacosta^28^	X	✓	✓	X	✓	✓	✓	5
24	Saleh^29^	✓	✓	✓	X	X	✓	✓	5
25	Esa et al.^30^	X	✓	✓	✓	✓	✓	✓	6
26	Thilander et al.^31^	X	✓	✓	X	✓	✓	✓	5
27	Freitas et al.^32^	X	✓	✓	X	✓	✓	✓	5
28	Bataringaya^33^	✓	✓	✓	✓	✓	✓	✓	7
29	Onyeaso^34^	X	✓	✓	X	✓	✓	✓	5
30	Tausche et al.^35^	✓	✓	✓	X	✓	✓	✓	6
31	Alhaija et al.^36^	X	✓	✓	X	✓	✓	✓	5
32	Ali and Abdo^37^	X	✓	✓	X	✓	✓	✓	5
33	Behbehani et al.^38^	X	✓	✓	✓	✓	✓	✓	6
34	Ciuffolo et al.^39^	✓	X	✓	X	✓	✓	✓	5
35	Karaiskos^40^	X	✓	✓	X	✓	✓	✓	5
36	Ahangar Atashi^41^	X	✓	✓	X	✓	✓	✓	5
37	Gelgör et al.^42^	X	✓	✓	X	✓	✓	✓	5
38	Jonsson et al.^43^	✓	✓	✓	✓	✓	✓	✓	7
39	Josefsson et al.^44^	X	✓	✓	X	✓	✓	✓	5
40	Ajayi^45^	X	✓	✓	X	✓	✓	✓	5
41	Mtaya^46^	✓	✓	✓	✓	✓	✓	✓	7
42	Borzabadi-Farahani et al.^47^	✓	✓	✓	X	✓	✓	✓	6
43	Daniel et al.^48^	X	✓	✓	✓	✓	✓	✓	6
44	Šidlauskas and Lopatienė^49^	X	X	✓	X	✓	✓	✓	4
45	Alhammadi^50^	✓	✓	✓	X	✓	✓	✓	6
46	Bhardwaj et al.^51^	✓	✓	✓	X	X	✓	✓	5
47	Nainani and Relan^52^	✓	✓	✓	X	X	✓	✓	5
48	Bugaighis et al.^53^	X	✓	✓	X	✓	✓	✓	5
49	Kaur et al.^54^	X	✓	✓	X	✓	✓	✓	5
50	Reddy et al.^55^	✓	✓	✓	X	X	✓	✓	5
51	Bilgic F et al.^56^	✓	✓	✓	X	✓	✓	✓	6
52	Gupta et al.^57^	X	✓	✓	X	X	✓	✓	4
53	Narayanan et al.^58^	✓	✓	✓	X	X	✓	✓	5

### Statistical analysis

Prevalence rates, by different variables, were presented as means and standard deviations (SD), with the minimum and maximum values. The data were checked for normal distribution using Kolmogorov-Smirnov test. As the distribution was not normal, analyses were conducted using non-parametric tests. Kruskal-Wallis test was used for comparisons between more than two groups. Mann-Whitney U test was used for pair-wise comparisons between groups whenever Kruskal-Wallis test was significant. Spearman's coefficient was calculated to determine the correlations, if any, between different variables. All tests were supposed to be two-tailed, and the power and the significance values were set at 0.8 and 0.05, respectively. Statistical analysis was performed with IBM^®^ SPSS^®^ Statistics for Windows software, version 21 (Armonk, NY: IBM Corp.)

## RESULTS

**Figure 1:**
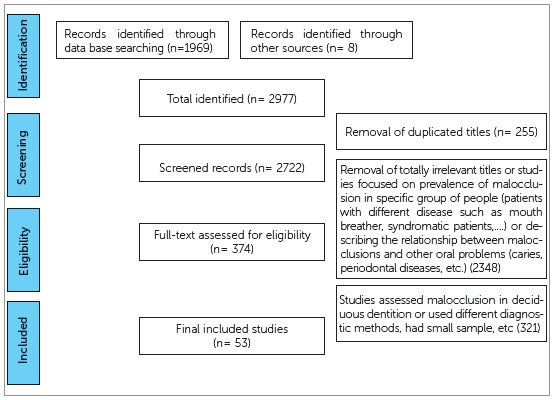
Flowchart of the literature selection process.

Two thousands nine hundreds and seventy seven studies were found to be potentially relevant to the study. The flow diagram (Fig 1) describes the process of articles retrieval; 255 articles were excluded due to duplication. The main cause of dropping of the retrieved articles was removal of irrelevant titles (2,348). The final closely related were 374 articles published between years 1951 and 2016. After reading their abstracts, only 53 articles (Table 1) fulfilled the inclusion criteria and were included in the subsequent analyses. 

The results of the critical appraisal of the included studies are presented in Table 2. The total quality score ranged from 4 to 7. Thirty eight studies (72%) were considered of moderate quality and fifteen (28%), of high quality. The most common drawbacks among all studies were failure to declare the study design (whether it is of cross-sectional, follow-up, etc.) and lack of sample size calculation.

In permanent dentition (Table 3), the global distributions of Class I, Class II, and Class III were 74.7%, 19.56% and 5.93%, respectively. Increased and reverse overjet was recorded in 20.14% and 4.56%, respectively. Regarding vertical malocclusions, the observed deep overbite and open bite were 21.98% and 4.93%, respectively. Considering the transverse occlusal discrepancies, the posterior crossbite affected 9.39% of the total examined sample.

**Table 3:** Global prevalence of malocclusion in permanent and mixed dentitions

Dimension	Malocclusion form	Permanent dentition				Mixed dentition			
		Min	Max	Mean	SD	Min	Max	Mean	SD
Antero-posterior	Class I	31	96.6	74.7	15.17	40	96.2	72.74	16
	Class II	1.6	63	19.56	13.76	1.7	58	23.11	14.94
	Class III	1	19.9	5.93	4.69	0.7	12.6	3.98	2.75
	Increased overjet	1.6	48.4	20.14	11.13	9.4	35.7	23.01	7.56
	Reversed overjet	0	20.1	4.56	5.26	0.4	11.9	3.65	3.67
Vertical	Deep bite	2.2	56	21.98	14.13	3.5	57.1	24.34	14.54
	Open bite	0.1	15	4.93	3.97	0.29	25.1	5.29	5.9
Transverse	Posterior crossbite	4	32.2	9.39	5.04	3.72	29.1	11.72	7.22

Regarding the distribution of malocclusion in adults according to geographical location (Table 4), four continents classification system was considered, in which Americas are considered as one continent. In permanent dentition, Europe showed the highest prevalence of Class II and posterior crossbite (33.51% and 13.8%, respectively), and the lowest prevalence of Class I (60.38%). This was applied to mixed dentition regarding Class I and Class II. No statistically significant differences in prevalence of Class III, increased overjet, reversed overjet, deep bite and open bite between the four geographic areas were reported. 

**Table 4:** Prevalence of malocclusion in different geographic locations.

Variable			Permanent dentition				P-value			
			America		Africa		Asia		Europe	
			Mean	SD	Mean	SD	Mean	SD	Mean	SD
Antero-posterior	Class I	78.53	8.56	83.68	12.48	78.93	9.77	60.39	16.76	0.019*
	Class II	15.25	7.06	11.45	9.08	12.26	4.28	33.51	17.73	0.016*
	Class III	6.23	2.68	4.75	4.6	6.32	6.46	6.2	2.75	0.5
	Increased overjet	16.67	5.61	21.4	13.91	19.79	10.5	20.79	12.38	0.9
	Reversed overjet	2.26	2.17	3.47	2.89	6.09	7	4.37	4.96	0.829
Vertical	Deep bite	11.13	6.41	25.83	18.96	23.83	12.95	21.56	13.33	0.227
	Open bite	5.03	4.32	6.34	3.12	4.01	3.86	4.92	4.82	0.378
Transverse	Posterior crossbite	7.08	2.24	7.9	1.78	8.27	2.65	13.08	7.93	0.029*
Mixed dentition										
Antero-posterior	Class I	69.98	19.67	90	6.11	72.78	10.29	63.95	13.77	0.035*
	Class II	27.22	20.22	7.5	5.71	21.42	10.4	31.95	12.47	0.024*
	Class III	2.78	0.84	2.48	0.59	5.76	3.91	3.53	1.21	0.226
	Increased overjet	21.12	8.23	21.23	11.3	25.09	7.62	23.02	5.12	0.841
	Reversed overjet	3.9	5.01	5.25	4.22	4.35	3.63	1.33	0.9	0.348
Vertical	Deep bite	14.98	7.73	23.3	15.5	22.09	9.97	37.4	17.62	0.122
	Open bite	5.57	3.09	8.3	5.31	4.5	7.79	4.18	5.79	0.077
Transverse	Posterior crossbite	10.67	8.26	12.13	6.62	17.77	8.47	12.45	6.54	0.832

*: Significant at P ≤ 0.05.

In permanent stage of dentition by ethnic groups, the highest prevalences of Class I malocclusion and open bite (89.44% and 7.82%, respectively) were reported among African population, although the difference of the latter was not statistically significant. However, the highest prevalence of Class II (22.9%) was reported among Caucasians. Otherwise, no statistically significant differences were found in prevalence of Class III, increased overjet, reversed overjet, deep bite and posterior crossbite between the three main populations (Table 5).

**Table 5:** Prevalence of malocclusion in different races

Variable			Permanent dentition				P-value	
			Africans		Caucasians		Mongoloids	
			Mean	SD	Mean	SD	Mean	SD
Antero-posterior	Class I	89.44	9.34	71.61	15.15	74.87	9.68	0.027*
	Class II	6.76	4.99	22.9	14.07	14.14	4.43	0.006*
	Class III	3.8	4.67	5.92	4	9.63	9.02	0.228
	Increased overjet	14.62	6.22	22.29	11.77	12.87	6.78	0.132
	Reversed overjet	3.5	2.93	3.99	5.11	10.87	6.68	0.122
Vertical	Deep bite	19.02	15.81	22.95	14.07	19.5	16.6	0.587
	Open bite	7.82	2.24	4.52	4.17	3.27	2.89	0.074
Transverse	Posterior crossbite	7.2	1.61	10.08	5.64	7.53	0.31	0.149
Mixed dentition								
Antero-posterior	Class I	92.47	4.41	70.39	14.78	66.75	1.77	0.02*
	Class II	5.1	3.8	25.91	14.86	22.1	0.85	0.028*
	Class III	2.4	0.69	3.53	1.86	10.95	2.33	0.045*
	Increased overjet	16.4	7.21	23.62	7.3	27.45	11.67	0.305
	Reversed overjet	3.9	3.97	3.15	3.59	8.5	1.77	0.217
Vertical	Deep bite	26.37	17.43	24.35	15.13	21.25	10.11	1
	Open bite	10	5	3.7	3.77	14.15	15.49	0.035*
Transverse	Posterior crossbite	10.77	7.39	11.64	7.49	16.2 (one case)		0.689

*: Significant at P ≤ 0.05.

The global distributions of Class I, Class II, and Class III in mixed dentition stage were 72.74%, 23.11% and 3.98%, respectively. The prevalence figures of increased and reverse overjet were 23.01% and 3.65%, respectively. Deep overbite and open bite cases were reported in 24.34% and 5.29%, respectively. Posterior crossbite represented 11.72% of the total pooled studies (Table 3). 

Regarding prevalence of malocclusion in mixed dentition according to geographical location (Table 4), Africa showed the highest prevalence of Class I (90%) but the lowest prevalence of Class II malocclusions (7.5%). The highest prevalence figures of Class II, Class III, and open bite malocclusions were reported in Europe (31.95%), Asia (5.76%), and Africa (8.3%), respectively. Deep bite was significantly higher in Europe (37.4%) compared to other geographical areas.

In mixed dentition, African population showed the highest prevalence of Class I (92.47%), but the lowest prevalence of Class II malocclusions (5.1%), while Caucasians showed the lowest prevalence of open bite (3.7%). Mongoloid showed significantly higher prevalence of Class III (10.95%). No significant differences in the prevalence of other malocclusions were found between different ethnicities (Table 5).

The prevalence of Class II was observed less frequently in permanent than in mixed dentition (19.56 ± 13.76 and 23.11 ± 14.94%, respectively), while the prevalence of Class III was observed more frequently in permanent than in mixed dentition (5.93 ± 4.96 and 3.98 ± 2.75, respectively).

## DISCUSSION

Global, regional and racial epidemiological assessment of malocclusions is of paramount importance, since it provides important data to assess the type and distribution of occlusal characteristics. Such data will aid in determining and directing the priorities in regards to malocclusion treatment need, and the resources required to offer treatment - in terms of work capacity, skills, agility and materials to be employed. In addition, assessment of malocclusion prevalence by different populations and locations may reflect existence of determining genetic and environmental factors. In line with that, the hypothesized tendency of changing prevalence of a specific type of malocclusion, such as Class II, from mixed to permanent dentition stage may give an indication about the effect of adolescent growth in correction of this problem. Finally, the availability of such global data will be important for educational purposes. Regional and/or racial-specific malocclusion may change the health policy toward developing the specialists’ skills and offering the resources required for that malocclusion. It must be emphasized that the current study summarizes the global distribution of malocclusion in mixed and permanent dentitions based on data extracted from studies of moderate (72% of the included studies) to high (28%) quality. None of the included studies was of low quality.

The pooled global prevalence of Class I was the highest (74.7 ± 15.17%), ranging from 31% (Belgium) to 96.6% (Nigeria). It was higher among Africans (89.44%), but equivalent among Caucasians and Mongoloids (71.61% and 74.87%, respectively). This pattern of distribution was reported for both dentitions with slight differences. Noteworthy, the prevalence of Class I in permanent dentition of Mongoloids tends to increase with pubertal growth, mostly due to the associated tendency for Class II correction in this race specifically.

The overall global prevalence of Class II was 19.56%. However, it was interesting to see a wide range from 1.6% (Nigeria) to 63% (Belgium). The lowest prevalence was reported for Africans 6.76% and the highest was reported for Caucasian (22.9%); the reported prevalence for Mongoloids was in-between (14.14%). The pattern of global distribution of Class II malocclusion by race was somewhat similar in mixed and permanent dentitions. With exception of African people (Africa), there is a tendency for correction of Class II with pubertal growth upon transition from mixed to permanent dentition. Both, prevalence and growth correction of Class II, can be attributed to the genetic influence. Recent research emphasizes the pivotal role of genetic control over condylar cartilage and condylar growth.^63^ ^,^ ^64^

The global prevalence of Class III was the lowest among all Angle’s classes of malocclusion (5.93 ± 4.69%). The range was interestingly wide: 0.7% (Israel) to 19.9% (China). The corresponding figures for Caucasians, Africans and Mongoloids were 5.92, 3.8% and 9.63%, respectively. This pattern of global distribution of Class III applies to mixed and permanent dentitions. A tendency to develop this type of malocclusion appears to increase upon transition from mixed to permanent dentition among Africans and Caucasians, rather than among Mongoloids. The role of genetics must be emphasized. In fact, Class III malocclusion in Asians is mainly due to the mid-face deficiency, rather than mandibular prognathism.^65^

The positive correlation found between Class II and increased overjet is logical. Simply, this is due to the fact that the most prevalent Class II malocclusion globally is Class II division 1.^66^ Similarly, the positive correlation of Class III malocclusion with reversed overjet is related to skeletal base discrepancy with minimal dentoalveolar compensation.^67^

The lowest prevalent malocclusion traits globally were reversed overjet and open bite (4.56 and 4.93, respectively). There is a high variation in prevalence of both traits as reported in the literature. Most of the studies reported that open bite trait is highly prevalent in African populations and low in Caucasian populations,^17^ ^,^ ^18^ ^,^ ^20^ ^,^ ^25^ in contrast to the reversed overjet, which reported to be prevalent in Mongoloids. In general, both traits are genetically determined.^63^ ^,^ ^64^

An interesting finding was the higher prevalence of Class II malocclusion in the mixed dentition than in the permanent dentition. This could be explained by the fact that self-correction of a skeletal Class II problem might occur in the late mixed and early permanent dentition stage as a result of a potential mandibular growth spurt. However, a sound conclusion can’t be drawn, as the present study was not prospective. In addition, the difference in leeway space between maxillary and mandibular arches, and residual growth in the permanent dentition stage could explain the higher prevalence of Class III malocclusion in the permanent dentition than in the mixed dentition, and the fact that the mandible might continue to grow till the mid- twenties. 

The present pooled data showed a decrease in the prevalence of deep bite upon transition from mixed to permanent dentition. Thilander et al,^31^ likewise, showed that increased overbite was more prevalent in the mixed dentition. Such an overbite reduction from the mixed to the permanent dentition is due to both occlusal stabilization involving full eruption of premolars and second molars, and the more pronounced mandibular growth.^35^ This also explains the reduction in Class II cases as well as the increase in Class III cases (reverse overjet as well) during the period of changing dentition. 

In addition to the importance of reporting global malocclusion, it is of an equal importance to report the worldwide orthodontic treatment needs. We planned to do so if the included studies had covered both issues. This was not the case, however, and hence we recommend addressing this latter issue with a similar systematic review.

## CONCLUSIONS

1) Consistent with most of the included individual studies, Class I and II malocclusions were the most prevalent, while Class III and open bite were the least prevalent malocclusions.
2) African populations showed the highest prevalence of Class I and open bite malocclusions, while Caucasian populations showed the highest prevalence of Class II malocclusion.
3) Europe continent showed the highest prevalence of Class II among all continents.
4) Class III malocclusion was more prevalent in permanent dentition than mixed dentition, conversely finding for Class II, while all other malocclusions variables showed no difference between the two stages.
